# Cloning, Expression, and Purification of Recombinant Lysostaphin From *Staphylococcus simulans*

**DOI:** 10.5812/jjm.10009

**Published:** 2014-05-01

**Authors:** Leila Farhangnia, Ehsanollah Ghaznavi- Rad, Neda Mollaee, Hamid Abtahi

**Affiliations:** 1 Department of Biotechnology, Arak University of Medical Sciences, Arak, IR Iran; 2 Department of Microbiology and Immunology, School of Medicine, Arak University of Medical Sciences, Arak, IR Iran; 3 Department of Biotechnology, School of Medicine, University of Medical Sciences, Arak, IR Iran; 4 Molecular and Medicine Research Center, Arak University of Medical Sciences, Arak, IR Iran

**Keywords:** *Escherichia coli*, Lysostaphin, Recombinant Proteins, *Staphylococcus*

## Abstract

**Background:**

*Staphylococcus aureus* is one of the most common causes of nosocomial infections and its resistance to antibiotics is a global concern. Lysostaphin is an antimicrobial agent belonging to a major class of antimicrobial peptides and proteins known as the bacteriocins. It exhibits a high degree of anti-staphylococcal bacteriolytic activity.

**Objectives:**

In this study, high level of recombinant mature lysostaphin in *Escherichia coli* was produced by using pET32a expression vector.

**Materials and Methods:**

The *S. simulans* gene encoding lysostaphin was extracted, amplified by polymerase chain reaction (PCR), and sub-cloned in prokaryotic expression vector pET32a. *E. coli *BL21 (DE3) plysS were transformed with pET32a-lys and gene expression was induced by IPTG. The expressed protein was purified by affinity-chromatography using (Ni-NTA) resin.

**Results:**

PCR and sequencing results confirmed the successful cloning of the target gene into the vector. The expression of protein was induced by IPTG and high concentration of the recombinant protein was obtained via the purification process by affinity-chromatography.

**Conclusions:**

Our data showed that the recombinant mature lysostaphin protein produced by pET32a vector in *E. coli* system was very efficient.

## 1. Background

*Staphylococcus aureus* is a major cause of both nosocomial and community-acquired infections worldwide that causes a wide range of diseases including endocarditis, osteomyelitis, pneumonia, toxic-shock syndrome, food-poisoning, carbuncles, and boils (1). Increased emergence of multidrug resistance among methicillin-resistant *S. aureus* (MRSA) strains has become a major concern in the hospital environment as it imposes a tremendous financial burden and increased morbidity and mortality due to hard-to-treat systemic infections (2).

Nowadays vancomycin is the antibiotic of choice for treatment of MRSA; however, accumulating mutations in *S. aureus* have led to intermediate resistance to vancomycin (VISA) (3). It has left us with the spectrum of very few effective antibiotics being available to treat *S. aureus* infections and with the probability that resistance to the remaining antibiotics would likely occur. Therefore, The issue of drug resistance in this group of pathogens needs to be addressed via appropriate use of existing drug as well as the development of novel agents (4, 5). Staphylococci are potential targets for bacteriocins including lysostaphin.

Lysostaphinis a glycine-glycine endo peptidase produced by *S. simulans *that specifically cleaves the glycine-glycine bond unique to the inter peptide cross-bridge of *S. aureus* cell wall. Due to its unique specificity, lysostaphin has a high potential for treating antibiotic-resistant Staphylococcal infections (1). Studies on the secondary protein structure of lysostaphin have revealed three distinct regions in the precursor protein: a typical signal peptide (ca. 38 amino acids), a hydrophilic and highly ordered protein domain with 14 repetitive sequences (296 amino acids), and the hydrophobic mature lysostaphin (6). Mature lysostaphin is a single polypeptide chain with molecular weight of 27 KDa (7).

Lysostaphin is unique in possessing extremely high activity against a variety of staphylococcal strains including MRSA. *S. aureus* is one of the most prevalent microorganisms of skin flora and coding sequence of the mature mostly isolated from wound, skin, and soft tissue infections. Therefore, production of the pure and effective recombinant lysostaphin (r-lysostaphin) protein *in vitro* could lead to a potential treatment for *S. aureus*. Previously, lysostaphin has been cloned and expressed heterologously in *Escherichia coli* (8), in the simian kidney cell line (9), and in *Lactococcus lactis* (10). At present, several lysostaphin overexpression systems are described (9-11). Although researcher have tried to improve the amount of lysostaphin production in these studies, the yield has remained unsatisfactory and purification methods are complicated.

## 2. Objectives

In the present study, we described a new expression system for producing r-lysostaphin in *E. coli,* a safe non-pathogenic host, on a laboratory scale with potentials to industrial scale. In addition, high-capacity Ni-NTA resin purification procedure was used for obtaining large amounts of pure lysostaphin.

## 3. Materials and Methods

### 3.1. Bacterial Strains, Vectors and Other Regents

*S. simulans *PTCC 1442 (Iran) and pET32a vector (Novagene, USA) was purchased. This vector is able to express a fusion protein with a 6-histidine tag at thrombin site and a T7 tag at the N-terminus. These additional amino acids increase the size of expressed protein near 15 KDa. Restriction enzymes, DNA ligase (Fermentas, Lithuania), were obtained. *E. coli* strain DH5α (f-gyr A96 Nalr, recA1 relA1 Thi-1 hsdR17 r-k m+k, Stratagene, USA) were used for initial cloning. The recombinant pET32a (pET32a- lysostaphin) was transformed into *E. coli*, BL21 (DE3) pLysS (f–ompthsdB, rB- mB-, dcm gal, DE3, pLYsScmr) as host strain from (Novagene, USA). 

Protein Purification Kit (Qiagene, Germany) were provided. MHB (Muller Hinton Broth, Sigma, USA) and LB broth (Luria Bertani Broth, Sigma, USA) were used for routine bacterial culture. The required antibiotics, ampicillin (100 μg/mL) and chloramphenicol (34 μg/mL) (Sigma, USA), were added to LB media according to the reference recommendation (12). All chemicals were obtained from Merck (Germany) and the enzymes were obtained from Fermentas (Lithuania) or Cinagene Tehran, (Iran) Companies.

### 3.2. Isolation of Plasmid DNA

After overnight incubation of *S. simulans *in MHB at 37°C, bacterial cells were centrifuged at 4000rpm for 5min and the pellet was suspended in 100 μL of SET buffer (sucrose 50 mM, EDTA 10 mM,Tris-HCl 25 mM, pH = 8). Plasmid DNA was prepared according to standard mini-preparation of plasmid Method. Briefly, bacterial pellet was obtained from 1.5 mL overnight bacterial culture re suspended in SET buffer and left at room temperature for 5 min. Afterwards, the bacterial cells were lysed by fresh, cold lyses buffer(NaOH5N, SDS 10%, DDW), then KAC (KAC 5M, Acetic acid, DDW) was added. 

The cell debris, proteins, and chromosomal DNA were removed by two times phenol/chloroform/isoamyl alcohol (25:24:1) Mixture. DNA was precipitated by ethanol (100%) and washed in ethanol (70%); then, the pellet was dried on air and resuspended in TE buffer. The quality and quantity of purified plasmid DNA were assayed by 0.8% Agarose gel electrophoresis in 1X TBE buffer and spectrophotometry(260/280 nm), respectively (12).

### 3.3. Polymerase Chain Reaction and Construction of the Recombinant Plasmid pET-lys

Primers were designed according to the full-length of lysostaphin gene sequence (Gene Bank accession no: X06121). The coding sequence of the mature peptide (738 bp) was amplified by polymerase chain reaction (PCR) using the following primers: 5´-AGA GGA TCC GCT GCA ACA CAT GAA -3´ (forward primer with an endonuclease site *Bam*HI) and 5´-CGC CTC GAG TCA CTT TAT AGT TCC-3´ (reverse primer with an endonuclease site *Xho*I). Restriction endonuclease sites for *Bam*HI and *Xho*I were incorporated at the 5´-and 3´-end of the mature gene, respectively, for sub-cloning purposes. Gene amplification of lysostaphin gene was performed in a total volume containing 20 ng of template DNA, 0.5 μM of each primers, 2 mM Mg2+,200 mΜ of each deoxynucleotide triphosphate,1X PCR buffer, and 2.5 unites of Taq polymerase. 

The following program (Eppendorf PCR) were used for amplification: Hot start at 94°C for five minutes, followed by 30 cycle of denaturation at 94°C for one minute, annealing at 56°Cfor one minute, and extension at 72°Cfor one minute. The program followed by a final extension at 72°C for five minutes. The PCR products were analyzed in 0.8% agarose gel in 1X TBE buffer and purified from gel by High Pure PCR product purification kit (Roche Diagnostics GmbH, Mannheim, Germany) according to the manufacturer’s instruction.

The PCR product was digested with *Bam*HI and *Xho*I and was ligated to pET32a, which were digested by the same restriction enzymes, to generate the recombinant plasmid pET32a-lys (pET-lys) using T4 DNA ligase (12). The *E. coli* DH5α was used for transformation of pET32a-lys plasmid. The transformed bacteria were selected by screening the colonies on Ampicillin (100 μg/mL) containing media and plasmid purification. Then colonies were further analyzed by restriction enzyme digestion and PCR. The lysostaphin gene of the recombinant plasmid was sequenced by Sanger method.

### 3.4. Expression and Purification of Recombinant Mature Lysostaphin

The expression host *E. coli* BL21 (DE3) pLysS was used as transformation host for pET-lys vector. This strain, containing T7 RNA polymerase gene under the control of lacUV5 promoter, was transformed with pET-lys. A single colony of transformed *E. coli* BL21 (DE3) with pET-lys was incubated overnight on shaking incubator in 2 mL Luria-Bertani broth (LB) medium containing Ampicillin (100 μg/mL) and chloramphenicol (34 μg/mL) at 37°C with constant agitation (200 rpm). The next day, 500 μL of cultured materials was removed and inoculated in 25 mL LB broth (per liter: 14 g yeast extract,12 g Bactotryptone, 10 g NaCl, 1 g KCl, 0.5 g MgCl, 0.5 g CaCl).

The culture was grown in an OD600nm of 0.6 with vigorous shaking (200 rpm) at 37°C. Isopropyl-β-D-thiogalactopyranoside (IPTG) was added to a final concentration of 1 mM for expression of mature lysostaphin in *E. coli*. The incubation period continued for another four hours at 37°C with shaking at 200 rpm. In order to produce the expression protein, bacterial suspension were tested at two and four-hour intervals and analyzed on 12% SDS-PAGE (13). The expressed protein was purified using Ni-NTA agarose column according to manufacturer’s instruction (Qiagene, Hilden, Germany).The purified protein was dialyzed and refolded with PBS (containing PMSF 0.2mM, pH = 7.2) at 4°C overnight. The quality and quantity of purified recombinant mature lysostaphin was analyzed on a 12% SDS-PAGE gel electrophoresis with Bradford methods (14).

## 4. Results

### 4.1. Isolation of Plasmid

The plasmid DNA of *S. simulans* was extracted and the concentration was adjusted to 4 μg/μL that were used as template for amplification of the gene encoded lysostaphin.

### 4.2. Construction of the Recombinant Plasmid pET-lys

The sequencing result was confirmed by comparing to database using basic local alignment search tool (BLAST) software. Enzyme digestion procedure, PCR assay, and sequencing result showed that target gene was inserted correctly into the recombinant plasmid pET-lys (data are not show).

### 4.3. Expression and Purification of Recombinant Mature Lysostaphin

**Figure 1.**
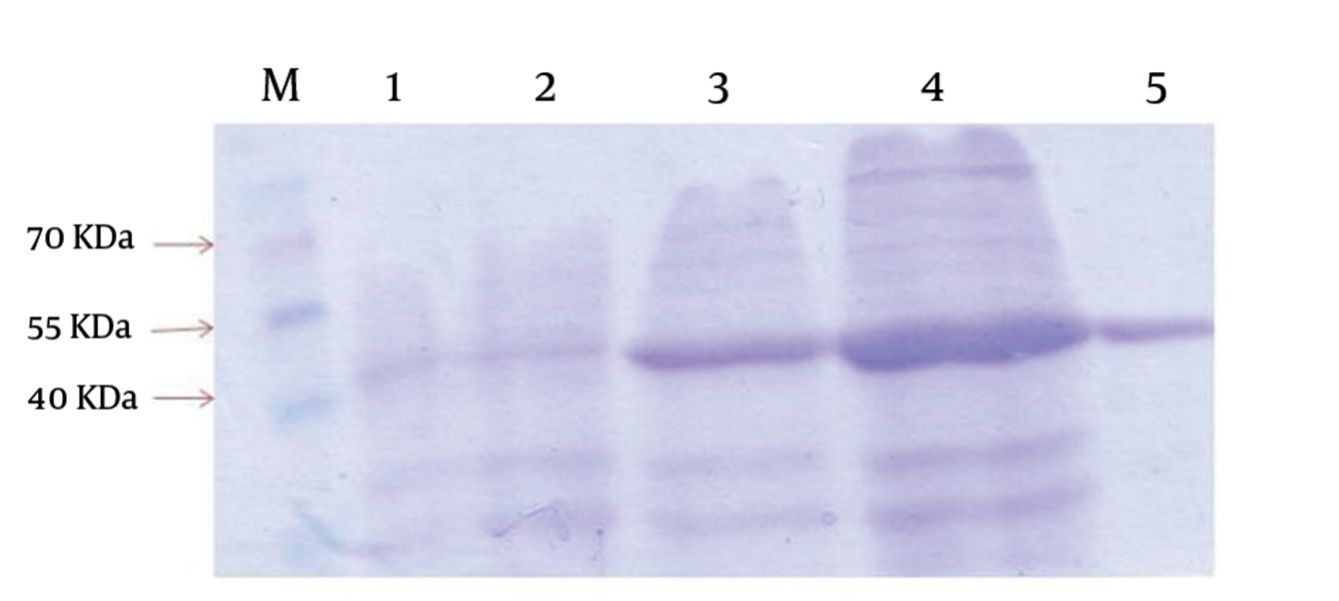
SDS-PAGE Gels With Coomassie Brilliant Blue Staining Showing the Expression and Purification of Recombinant Lysostaphin in pET 32a System The SDS-PAGE gels shows uninduced cell extract from *E. coli* BL21(DE3)+PET-lys for one hour(lane 1) and two hours (lane 2), induced cell extract for two hours(lane 3) and four hours (lane4), and Extracted Proteins after Ni-NTA affinity chromatography (lane 5). A high-range molecular weight Marker is shown on left (lane M).

The positive recombinant plasmid was transformed into the host, *E. coli* BL21 (DE3). The addition of IPTG induced the overexpression of approximately 42 kDa molecular weight recombinant protein. The expressed protein was purified successfully via affinity chromatography using Ni-NTA resin (Figure 1). The purification and dialysis process resulted in the yield of about 30 mg of purified protein from 1 L of *E. coli* BL21 (DE3) + pET-lys culture.

## 5. Discussion

In this study, the mature lysostaphin recombinant protein from *S. simulans* was cloned, expressed under the control of T7 promoter, and purified using Ni-NTA resin. The obtained results showed that pET 32a system was very efficient.

Unlike an antibiotic, which interferes with bacterial growth, lysostaphin is highly effective in lysing *S. aureus* cells throughout the metabolic stage. Earlier methods for production of lysostaphin endopeptidase aimed to purify it from crude extract of *S. simulans *(15, 16), which might be contaminated with small amounts of pyogens/allergens. In addition, mature lysostaphin is cleaved off the propeptide again using *S. simulans* extract (9, 17). However, purification of wild-type lysostaphin is very difficult. Although several methods of lysostaphin production have reported, the yield and purity were very limited (11, 17, 18).

There are a number of reports for expression of lysostaphin endopeptidase in *E. coli* using the lysostaphin endopeptidase promoter (6, 8). Proendopeptidase was also expressed in eukaryotic system under the transcriptional control of Cytomegalovirus (CMV) promoter (9). The expression of recombinant prolysostaphin in *Bacillus subtilis* and *B.* *sphaericus *was reported that revealed their ability to secret large amount of lysostaphin into the culture medium. *B. sphaericus* produces about five times more lysostaphin than its natural source (19). 

Lysostaphin was also expressed in mice, in which the 5′-flanking region of the Bovine β-lactoglobulin gene directed the secretion of lysostaphin into milk (20). As far as pharmaceutics/therapeutics are concerned, *E. coli* is considered a safe expression host. Numerous proteins have been expressed in *E. coli*; therefore, *E. coli* is widely used as an expression host in both research and industry.

In several study, r-lysostaphin were produced through different pET vectors including pET28a with the yield of 22 mg, pET 23b with the yield of 20 mg, and pET15b with the yield of 11 mg of purified protein from 1 L of *E. coli* BL21(DE3) + pET-lys culture (7, 21, 22). The lysostaphin was also overexpressed and purified using the intein–chitin-binding domain (intein–CBD) as a fusion protein with the yield of 6 mg/L (11). A r-lysostaphin expressed in *E. coli* is sold commercially by Sigma-Aldrich and is indispensable for Staphylococcal genetic studies; it is used for DNA isolation (23), formation of protoplasts, and differentiation of Staphylococcal strains (24). 

Further evaluation of the anti-Staphylococcal potential of lysostaphin as a therapeutic agent and its use as a laboratory reagent depends on the availability of large amounts of highly purified protein from a safe and nonpathogenic source. Therefore, the low yield obtained in lysostaphin production (7, 21, 22), pathogenesis, and multi-drug resistance properties of *S. aureus *(2) as well as high-cost industrial product of lysostaphin have been the principal reason to search for a recombinant source for this therapeutic agent. This is the first report of recombinant mature lysostaphin from Iran.

In the present study, pET32a system was used to express the r-lysostaphin in *E. coli*. Using this purification method, we obtained about 30 mg of r-lysostaphin per liter of the growth medium in the pET 32a system. In this assay, the r-lysostaphin was purified using Ni-NTA column according to manufacturer’s instruction (Qiagene, Germany). This purification method is very simple and was performed in laboratories that had neither the expertise nor the equipment necessary for traditional protein purification schemes. The procedure for producing r-lysostaphin is quite convenient and efficient and would allow a laboratory to produce large amounts of r-lysostaphin. In this study, in order to obtain high level expression of fusion proteins, *E. coli* BL21 (DE3) plys S was used as a expressed host that is deficient in the known cytoplasmic protease gene products (25). Therefore, the highest expression of lysostaphin in *E. coli *BL21 (DE3) plys S might be due to protease deficiency in this strain. The pET system has been recognized as one of the most powerful methods for producing recombinant proteins in *E. coli* and the significant advantages of this system have been widely discussed.

Therefore, we produced mature r-lysostaphin with the presented procedure from *E. coli* for preparation of large quantity of r-lysostaphin for structure function studies and evaluation of its clinical potential in therapy as well as prophylaxis against staphylococcal infections. Our data showed that mature lysostaphin region of lysostaphin gene can be expressed by pET32a vector in *E. coli*, and T7 lac promoter might be stronger than other promoters in inducingr-lysostaphin production.
